# Response to Matters arising: comments on “Radiological risk factors in different patterns of patellar instability”

**DOI:** 10.1186/s43019-026-00323-7

**Published:** 2026-08-11

**Authors:** Devendra Kumar Chouhan, Supreeth Kumar

**Affiliations:** Department of orthopaedic surgery, Post Graduate Institute of Medical Education and Research, Chandigarh, India

Dear Editor,

We sincerely thank the authors of the Letter to the Editor for their thoughtful and comprehensive critique of our article, “Radiological risk factors in patellar instability: a comparative analysis of single-episode, recurrent, and habitual patella dislocation” [1]. Their insightful observations regarding methodological rigor and clinical translatability have provided us with a valuable opportunity to clarify our study’s design, address specific concerns, and discuss the broader implications of our findings. We appreciate the constructive nature of their commentary.

## Point-by-point response to raised concerns

### Statistical independence and bilateral case clustering

The concern regarding statistical independence in bilateral cases is well-founded and represents a recognized challenge in orthopedic research involving paired anatomical structures [2]. There are methodological justifications for our study design, as bilateral involvement may demonstrate asymmetric severity and risk factor profiles, even within the same patient. Each knee represents a distinct clinical entity requiring individual assessment for surgical planning [3]. Excluding bilateral cases or arbitrarily selecting one knee would reduce statistical power and potentially introduce selection bias. The primary objective was to characterize radiological phenotypes across instability patterns, where each knee contributes unique anatomical information.

However, potential inflation of statistical significance is valid. To address this, we conducted a post hoc sensitivity analysis using only one knee per patient (randomly selecting one knee from bilateral cases), reducing our sample to 106 knees. The key findings remained consistent; trochlear dysplasia maintained significant association with instability type (*p* = 0.005, Cramér’s *V* = 0.31), demonstrating moderate strength of association. Tibial tubercle–trochlear groove (TT–TG) distance continued to show progressive increase across groups (single patellar dislocation [SPD]: 18.2%; recurrent patellar dislocation [RPD]: 31.4%; habitual patellar dislocation [HPD]: 75%; *p* = 0.008). Correlation between severity and cumulative risk factors remained significant (*p* = 0.002).

These results confirm that our primary conclusions are robust even when accounting for within-patient clustering. The slightly adjusted *p*-values remain statistically significant, suggesting that our original findings were not artificially inflated by the inclusion of bilateral cases.

### Selection and spectrum bias

We excluded patients who had undergone previous patellar stabilization and did not have relevant preoperative radiological records. Postsurgical anatomical alterations (e.g., medialization of tibial tubercle, trochleoplasty, or soft tissue reconstruction) would fundamentally alter the radiological measurements and add to the heterogeneity. Moreover, our objective was to characterize native anatomical risk factors, not postintervention anatomy.

Our findings should therefore be interpreted as characterizing the radiological phenotype of non-operatively managed SPD without any reported episode of recurrence compared with RPD and habitual patellar dislocation (HPD). This actually enhances clinical utility, as these are the patients for whom radiological risk stratification most influences management decisions.

### Measurement reproducibility and observer reliability

We employed several strategies to minimize measurement variability:

1. All measurements were performed by two experienced musculoskeletal radiologists (with 8 and 12 years of experience) using a standardized protocol.
2. Images were selected by consensus between two observers and saved; only those were used for further measurement to reduce image selection bias.
3. Measurements were made independently, and discrepancies > 2 mm for continuous measures or disagreements in categorical classifications were resolved through consensus review.
4. We utilized validated measurement techniques (Pfirmann’s method for TT–TG, standard sagittal technique for Caton Deschamps (CD) ratio, and Dejour classification for dysplasia)
5. All measurements were performed using the same DICOM viewer software with standardized windowing.

However, we strongly advocate for prospective studies to incorporate formal reliability assessment as an integral component of study design, with sample size calculations that account for both the primary research question and reliability estimation.

### TT–TG distance: discriminability and clinical actionability

We welcome the opportunity to provide additional analyses that enhance clinical actionability. Therefore, we performed threshold-based analysis from previously saved images (Table 1).

**Table 1:** Threshold-based analysis

Group	> 15 mm	> 20 mm	Mean ± standard deviation (SD)
SPD (*n* = 39)	7 (17.9%)	1 (2.6%)	11.69 ± 3.6 mm
RPD (*n* = 73)	24 (32.9%)	4 (5.5%)	13.81 ± 2.64 mm
HPD (*n* = 12)	9 (75.0%)	5 (41.7%)	19.37 ± 8.52 mm
Chi-squared analysis	*p* < 0.001	*p* < 0.001	
Cramér’s *V*	0.31 (moderate association)	0.38 (moderate association)	

We acknowledge the small mean difference between SPD and RPD; a 2.12 mm mean difference, while statistically significant, may not represent a clinically meaningful distinction for individual patient management. The substantial overlap in distributions (as reflected in the standard deviations) supports this concern.

However, we offer several contextual considerations:

1. Population-level versus individual-level interpretation: Our findings describe population-level phenotypic differences rather than diagnostic thresholds for individual patients. The progressive increase in mean TT–TG across instability patterns (SPD → RPD → HPD) suggests a dose–response relationship between lateralization and instability severity.
2. Threshold exceedance is more clinically relevant: As the author correctly emphasized, the threshold-based analysis provides more actionable information. The prevalence of TT–TG >15 mm approximately doubled from SPD (17%) to RPD (32%) and more than doubled again from RPD (32%) to HPD (75%). We agree entirely with the “menu à la carte” concept [4]. TT–TG should not be interpreted in isolation but rather as one component of a multifactorial anatomical construct [5].

Multifactorial risk assessment: To address the concern about isolated TT–TG interpretation, we provide an expanded analysis examining TT–TG in the context of co-factors:

This stratified analysis demonstrates that elevated TT–TG is relatively uncommon in SPD patients with normal trochlear morphology (5.0%). The combination of high-grade dysplasia and elevated TT–TG is progressively more common with increasing instability severity. This supports the multifactorial model where anatomical risk factors interact synergistically (Table 2).

**Table 2:** TT–TG > 15 mm stratified by trochlear dysplasia

Group	Normal trochlea	Type A dysplasia	High-grade dysplasia (B/C/D)
SPD	1/20 (5.0%)	2/6 (33.3%)	4/13 (30.8%)
RPD	3/25 (12.0%)	5/13 (38.5%)	16/35 (45.7%)
HPD	0/0 (N/A)	0/0 (N/A)	9/12 (75.0%)

### Clinical actionability

For SPD patients: TT–TG > 15 and 20 mm is present in only 17.9% and 2.6% of cases, respectively, suggesting that lateralization is not the primary driver in most first-time dislocations substantiating observation of Wierer et al. [6]. When present, elevated TT–TG is usually accompanied by other risk factors (particularly high-grade dysplasia). This also supports selective consideration of stabilization for SPD in only those with multiple risk factors [5].

TT–TG > 15 mm is present in 32.9% and 75% and > 20 mm in 5.5% and 41.7% in RPD and HPD groups, respectively.

### Construct validity: inclusion of habitual patellar dislocation

This represents perhaps the most conceptually important critique, and we appreciate the opportunity to clarify our rationale for including HPD while acknowledging the validity of the concerns raised.

Rationale for inclusion: Our decision to include HPD in the comparative analysis was based on several considerations:

1. Spectrum of disease: We conceptualized patellar instability as existing along a severity spectrum, with HPD clinically and radiologically representing the most severe category rather than a distinct entity. This framework allowed us to examine whether anatomical risk factors show dose–response relationships with clinical severity.
2. Comprehensive phenotyping: Including HPD provided the opportunity to characterize the extreme end of the anatomical spectrum, which may inform our understanding of pathomechanics across all instability patterns.
3. Limited existing data: Comparative studies including HPD are relatively scarce in literature, and we felt that comprehensive phenotyping of this challenging patient population had value.

We recognize the validity of the concerns:

1. Etiologic distinction: HPD often represents a developmental condition, which starts very early, often in the first decade of life, and with fundamentally different pathogenesis. The anatomical abnormalities in HPD are not merely “risk factors” but might constitute the structural substrate of the condition itself.
2. Management extrapolation concerns: The surgical principles for HPD (comprehensive multiplanar reconstruction) differ substantially from those for episodic instability (selective correction of specific anatomical factors). Presenting these conditions in the same comparative framework could invite inappropriate extrapolation.

### Sensitivity analysis excluding HPD

To address these concerns, we performed sensitivity analyses examining only 112 SPD and RPD knees (episodic instability). After excluding HPD, the associations between anatomical risk factors and instability type become substantially weaker and lost statistical significance for most parameters. This suggests that:

1. The strong associations reported in our original analysis were largely driven by the inclusion of HPD cases.
2. The anatomical distinctions between SPD and RPD are more subtle than the distinctions between episodic instability and HPD.
3. SPD and RPD may represent different points along a continuum rather than categorically distinct phenotypes (Table 3).

**Table 3:** Sensitivity analysis of SPD and RPD

Parameter	SPD (*n* = 39)	RPD (*n* = 73)	*p*-value	Cramér’s *V*
High-grade dysplasia	13 (33.3%)	35 (47.9%)	0.118	0.15 (weak)
TT–TG > 15 mm	7 (17.9%)	24 (32.9%)	0.081	0.16 (weak)
Patella alta	20 (51.3%)	32 (43.8%)	0.443	0.07 (weak)
≥ 2 risk factors	14 (35.9%)	31 (42.5%)	0.486	0.07 (weak)

In light of this sensitivity analysis and the comment from the author, it would be better to have a two-tiered model:

Tier 1: Episodic instability (SPD and RPD) represents a continuum of traumatic or atraumatic instability, anatomical factors function as risk modifiers that influence recurrence probability, and anatomical distinctions between SPD and RPD are modest and overlapping; therefore, management decisions should be individualized on the basis of multifactorial risk assessment rather than simple SPD versus RPD categorization [5–7]

Tier 2: Habitual dislocation (HPD) represents a distinct etiologic entity with developmental origins. Anatomical abnormalities are severe and universal, and constitute the bony and extensor mechanism structural basis of the condition. This requires comprehensive surgical reconstruction rather than selective anatomical correction [8]. It should not be conflated with episodic instability for management decision-making.

## Additional clarifications

### Power analysis and patella alta findings

The commentators implicitly raised concerns about our patella alta findings given the lower statistical power (0.51) for this parameter. We acknowledge this limitation explicitly. Our post hoc power analysis revealed adequate power (> 0.80) for detecting moderate associations with trochlear dysplasia and TT–TG distance, but lower power (0.51) for patella alta. This reflects the smaller effect sizes observed for patella alta. The weak and nonsignificant associations we observed for patella alta should be interpreted cautiously. Our study was underpowered to definitively conclude that patella alta is not associated with instability patterns. Rather, we can conclude that:

1. If an association exists, it is weaker than those for trochlear dysplasia and TT–TG distance.
2. Our sample size was insufficient to detect small-to-moderate associations.
3. The unexpected pattern (higher prevalence in SPD than HPD) warrants further investigation in larger cohorts.

However, the role of patella alta in patellar instability remains debated in literature, with some studies showing strong associations [9, 10] and others showing weak or inconsistent relationships [6, 11]. Our findings contribute to this mixed evidence base but should not be overinterpreted given the power limitations.

## Concluding remarks

We are genuinely grateful to the authors of the Letter to the Editor for their rigorous and constructive comments. Their observations have prompted us to conduct additional analyses that strengthen and refine our conclusions, and have highlighted important considerations for future research in this field.

On the basis of our original analysis and the additional analyses conducted in response to this critique, we offer the following refined conclusions:

1. Trochlear dysplasia represents the most consistent and strongest anatomical risk factor across the spectrum of patellar instability, with progressive severity from SPD to RPD to HPD.
2. TT–TG distance shows a progressive increase across instability patterns, with threshold exceedance (> 15 mm and > 20 mm) providing more clinically actionable information than mean differences. The modest difference between SPD and RPD suggests overlap in anatomical phenotypes.
3. Patella alta shows weak and inconsistent associations with instability patterns in our cohort, though our study was underpowered to detect small effects. Its role requires further investigation in larger studies.
4. Cumulative risk factors show a strong association with instability severity, supporting multifactorial risk assessment approaches.
5. Habitual patellar dislocation should be conceptualized as a distinct etiologic entity rather than simply the severe end of an episodic instability spectrum, with implications for both research design and clinical management.

We hope that this detailed response addresses the concerns raised and contributes to the ongoing scholarly dialogue regarding radiological risk factors in patellar instability. We welcome continued discussion and look forward to future collaborative efforts to advance understanding in this important clinical area.

## Data Availability

No datasets were generated or analyzed during the current study.
